# Computational Design of Potentially Multifunctional Antimicrobial Peptide Candidates via a Hybrid Generative Model

**DOI:** 10.3390/ijms26157387

**Published:** 2025-07-30

**Authors:** Fangli Ying, Wilten Go, Zilong Li, Chaoqian Ouyang, Aniwat Phaphuangwittayakul, Riyad Dhuny

**Affiliations:** 1 Department of Computer Science and Engineering, State Key Laboratory of Bioreactor Engineering, East China University of Science and Technology, Shanghai 200237, China; y30221685@mail.ecust.edu.cn (W.G.);; 2 International College of Digital Innovation, Chiang Mai University, Chiang Mai 50200, Thailand; aniwat.ph@cmu.ac.th; 3 Department of Creative Arts, Film and Media Technologies, University of Technology, Mauritius, La Tour Koenig, Pointe-aux-Sables 11134, Mauritius; dhuny@utm.ac.mu

**Keywords:** antimicrobial peptides, GANs, Deep Generative Models

## Abstract

Antimicrobial peptides (AMPs) provide a robust alternative to conventional antibiotics, combating escalating microbial resistance through their diverse functions and broad pathogen-targeting abilities. While current deep learning technologies enhance AMP generation, they face challenges in developing multifunctional AMPs due to intricate amino acid interdependencies and limited consideration of diverse functional activities. To overcome this challenge, we introduce a novel de novo multifunctional AMP design framework that enhances a Feedback Generative Adversarial Network (FBGAN) by integrating a global quantitative AMP activity regression module and a multifunctional-attribute integrated prediction module. This integrated approach not only facilitates the automated generation of potential AMP candidates, but also optimizes the network’s ability to assess their multifunctionality. Initially, by integrating an effective pre-trained regression and classification model with feedback-loop mechanisms, our model can not only identify potential valid AMP candidates, but also optimizes computational predictions of Minimum Inhibitory Concentration (MIC) values. Subsequently, we employ a combinatorial predictor to simultaneously identify and predict five multifunctional AMP bioactivities, enabling the generation of multifunctional AMPs. The experimental results demonstrate the efficiency of generating AMPs with multiple enhanced antimicrobial properties, indicating that our work can provide a valuable reference for combating multi-drug-resistant infections.

## 1. Introduction

The rapid increase in microbial resistance to conventional antibiotics represents a major global health challenge largely driven by the widespread misuse of chemical antibiotics [1]. Over the past decade, there have been several developments in utilizing AMPs as potential alternatives to treat infections, since most natural AMPs have broad-spectrum activity against a wide range of pathogens and are less prone to resistance development. Furthermore, many peptides exhibit distinct biological activities, play vital roles in the innate immune response across many organisms, and exhibit a range of antimicrobial activities against bacteria, fungi, viruses, and even cancer cells [2,3]. Their wide range of antimicrobial activities plays a key role in developing innovative treatments. Unfortunately, the vast sequence space of potential peptide candidates, coupled with the complex interplay between sequence, structure, and function, make traditional experimental approaches to AMP discovery time-consuming and resource-intensive [4]. In this context, the introduction of deep learning techniques provides a promising opportunity to speed up the discovery of multifunctional AMPs [5].

The utilization of deep learning methods for the development of new AMPs has garnered significant attention in recent years [5]. For instance, the implementation of Generative Adversarial Networks (GANs) [6,7,8,9] and Variational Autoencoders (VAEs) [10] has enhanced the efficiency of AMP generation, especially in combating pathogenic microorganisms, with broad-reaching applications in medicine and biology [11]. A notable study on the FBGAN [12] employs a feedback-loop in a GAN model to generate synthetic DNA sequences encoding proteins with antimicrobial properties. Through adversarial training, the ability of the generator in the FBGAN to create sequences resembling real peptides improves. The critical element in the FBGAN framework for optimizing AMP functionality is the classifier, which guides the training process towards generating AMP-like sequences. It assesses the sequences for AMP properties, where candidates with scores above 0.8 are included in the dataset, through a feedback mechanism for iterative improvement. However, the exact influence of the classifier on the quality and effectiveness of de novo peptide design remains a crucial question. This study aims to enhance the role of the classifier and its impact on improving the quality of newly designed peptides.

Despite recent advancements in data-driven generative models, challenges persist in the exploration of a wide range of AMPs with multifunctional activities, primarily due to the sparse distribution of AMPs across the entire peptide space. Thus, there is demand for accurate prediction of MIC values for efficient sampling and filtering of AMP analogues [13]. This involves combining a large dataset with a regression model to design AMPs with low MIC. Numerous studies have developed diverse databases containing experimentally validated AMPs and annotations of their functional activities. The third version of the Antimicrobial Peptide Database (APD3) includes AMPs with various functions, such as antibacterial, antifungal, antiviral, antiparasitic, and anticancer activities. It also offers a glossary, a classification section, a search function, and a prediction system [14]. Additionally, it provides a comprehensive analysis of AMPs and their antimicrobial activities against fungi and viruses.

Furthermore, some Deep Neural Network (DNN)-based prediction approaches have been proposed to predict AMPs with only a single specific type of functional activity, such as DeepAVP for predicting antiviral peptides [15], Deep-AFPpred for predicting antifungal peptides [16], and StaBle-ABPpred [17] and Deep-ABPpred [18] for predicting antibacterial peptides. However, these predictors have limited predictive capability and cannot provide comprehensive functional activity annotations of AMPs. Therefore, only a few predictors can predict multiple functional activities of AMPs [19], given the significance of AMP functional activities and the fact that no exiting studies have systematically summarized and evaluated these computational approaches for predicting AMPs and their functional activities. Additionally, some existing predictors are trained on imbalanced or incomplete datasets, with fewer than 50 negative samples and fewer than 5 positive samples; thereby, we assume that the predictive results of these models could be less reliable, given the fact that many publicly available AMP databases have been updated recently. In view of these shortcomings, it is necessary to curate a comprehensive training dataset by integrating the available databases and developing more accurate predictors for identifying AMPs and their multiple functional activities.

In recent studies, more advanced generative frameworks have emerged, including diffusion models and Protein Language Model (PLM)-based pipelines. For example, ProT-Diff integrates a pre-trained PLM with a diffusion model to generate structurally diverse AMPs from latent representations, decoupling sequence encoding and generation [20]. This approach enables control over peptide length and diversity, with high experimental success rates. Similarly, a quantum-annealing-based framework proposed by Tucs et al. combines a binary VAE with quantum annealing for multi-objective AMP design, demonstrating effective exploration of discrete latent spaces to optimize both antimicrobial activity and hemolyticity [21].

Moreover, BERT-AmPEP60 shows how Large Language Models (LLMs) like ProtBERT can be fine-tuned to predict MIC with high accuracy, leveraging transfer learning to address data scarcity in regression tasks [22]. Compared to traditional ML and shallow DL models, LLMs offer improved feature extraction from sequences alone, enhancing generalization across bacterial species.

In this paper, we introduce a two-stage framework for de novo design of multifunctional AMPs. This framework enhances the FBGAN by integrating two key modules: a global quantitative AMP activity regression module and a multifunctional-attribute integrated prediction module. In the first stage, by integrating a number of updated AMP databases, our model can be extensively trained to generate potential AMP candidates. The feedback-loop can significantly enhance the quality of resulting AMPs with a global quantitative AMP activity regression method. In the second stage, we employ a combinatorial predictor to simultaneously identify and predict five multifunctional AMP activities, enabling the accurate selection of multifunctional AMPs.

## 2. Results and Discussion

### 2.1. Implementation Details

#### 2.1.1. Data Gathering and Preprocessing

To support the training of key components in our framework, we curated two distinct datasets through systematic data processing. The first is a binary classification dataset, used within the FBGAN architecture to distinguish AMPs and non-AMPs. The second is a multifunctional prediction dataset, including peptides annotated with 5 core biological activities (antibacterial, antifungal, anticancer, antiviral and antiparasitic), employed to train the multifunction predictor.

For the binary classification task, we collected AMP sequences from four well-established AMP databases, GRAMPA [13], APD3 [14], ADAM [23], and CAMPR4 [24], along with additional AMP entries from UniProt. To complement this, non-AMP sequences were sampled from UniRef90 and supplemented with synthetically generated peptides matched in terms of their length and physicochemical properties. All sequences were filtered to exclude those with non-standard amino acids and clustered using CD-HIT to reduce redundancy. The final binary classification dataset comprised 14,021 AMPs and 58,775 non-AMPs, enabling effective training of the discriminator within the FBGAN framework.

For the multifunctional prediction task, we constructed a comprehensive dataset of 14,731 unique positive and 19,793 unique negative AMPs. The positive AMP sequences were aggregated from multiple sources, including APD3 [14], ADAM [23], CAMP [25], iAMP-2L [26], iAMP-CA2L [27], AMPfun [28], DRAMP [29], dbAMP [30], DBAASP [31], LAMP [32], ParaPep [33], phytAMP [34], AVPdb [35], CancerPPD [36], and milkAMP [37]. Sequences containing non-standard amino acids (e.g., B, J, O, U, X, Z) were removed to ensure high data quality. The negative dataset for this task consisted of peptides retrieved from UniProt, with all entries containing antimicrobial-related annotations (e.g., antibacterial”, “antiviral”, “antifungal”, “antiparasitic”, and “anticancer”) removed. Only sequences between 10 and 200 residues in length and composed of standard amino acids were retained. To minimize redundancy and eliminate potential overlap with the positive set, CD-HIT was applied using a 40% sequence identity threshold.

To enhance the consistency and interpretability of functional annotations, we consolidated 22 original functional labels derived from the study [19] into five biologically coherent core categories: antibacterial, antifungal, anticancer, antiviral, and antiparasitic. This reclassification was guided by biological relevance and functional similarity rather than introducing new labels, ensuring that the aggregated categories retained meaningful biological interpretability. Specifically, as shown in Table 1, the antibacterial category encompasses labels related to bacterial targeting, including antibacterial, anti-Gram-positive, anti-Gram-negative, anti-TB, and antibiofilm. The antifungal category integrates general antifungal activity and anticandida annotations. For anticancer activity, we merged labels such as anticancer and anti-mammalian-cell, which reflect activity against tumor- or host-derived cells. The antiviral category includes both general antiviral activity and specific anti-HIV annotations. Lastly, the antiparasitic category unifies labels targeting eukaryotic parasites, such as antiparasitic, antimalarial, antiplasmodial, and antiprotozoal. Less-common or ambiguous labels (e.g., insecticidal, non-anticancer cytotoxic, hemolytic) were excluded to avoid ambiguity and reduce noise in the functional annotation. This biologically driven reclassification reduced redundancy and improved the coherence of the label space, facilitating more robust and interpretable training of the multi-label classification model.

#### 2.1.2. GAN Training and Evaluation

The generator begins with a 128-dimensional random noise vector z, sampled from a Gaussian distribution. This vector is first passed through a fully connected layer to expand its dimensionality, preparing it for sequence generation. The transformed representation then flows through several residual blocks composed of 1D convolutional layers with skip connections, which facilitate learning of complex features while mitigating vanishing gradients. A final 1D convolutional layer shapes the output into a sequence of the desired length. To produce discrete amino acid representations, the output is passed through a Gumbel-Softmax layer implemented in PyTorch version 2.1.2, yielding a differentiable one-hot encoded sequence.

The discriminator, in contrast, is designed to distinguish between real and generated sequences. The input sequence is transposed and passed through a 1D convolutional layer with a 5 × 1 kernel to extract the initial features. It then goes through multiple residual blocks for further refinement. After feature extraction, the output is flattened and processed by a fully connected layer to yield a scalar output representing the authenticity score.

The GAN was trained using Adam optimizers for both the generator and discriminator with a learning rate of 0.0001 and decay rates of 0.5 and 0.9. For each training iteration, the discriminator was updated 10 times, incorporating a gradient penalty to enforce Lipschitz continuity, while the generator was updated once. The training was conducted for 80 epochs with a batch size of 64, using the Wasserstein loss function for both networks.

Throughout training, generator and discriminator losses were monitored to evaluate model convergence. Periodic sampling of the generated sequences allowed for qualitative inspection to verify that the generator was learning biologically meaningful patterns. Upon completion, the sequences were decoded from the one-hot format via argmax selection in each position and further analyzed for functional evaluation.

#### 2.1.3. Training and Evaluation of the Multifunction Predictor

The multifunction predictor employed in this study was built using a single-stage CNN framework. The model was designed to perform multi-label classification of AMP functional activities based on sequence information alone. It was trained on a comprehensive dataset comprising 14,731 positive and 19,793 negative AMP sequences with unique entries, where labels were determined based on the presence or absence of annotations for five distinct biological functions, including antibacterial, antiviral, antifungal, antiparasitic, and anticancer activity.

The dataset was randomly split into 80% for training and 20% for independent testing. To enrich the sequence representations, we applied multiple encoding strategies, including one-hot encoding, BLOSUM62 substitution matrices, AAIndex features, and PAAC encoding. These encoding schemes captured a diverse range of features such as amino acid composition, evolutionary conservation, and physicochemical properties, enabling the model to learn from both low-level and high-level sequence attributes.

Each of the five core AMP functionalities (antibacterial, antifungal, anticancer, antiviral, and antiparasitic) was modeled using an independent binary classifier. All five classifiers share the same underlying CNN architecture but are trained separately with their own dedicated datasets, each containing positive and negative examples specific to each function. This design allows each classifier to specialize in learning sequence and structural features unique to its assigned activity, enhancing its task-specific predictive accuracy.

Each independent classifier was trained for 100 epochs using the Adam optimizer with a batch size of 32. The initial learning rate was set to 0.0005 and halved every 10 epochs to ensure stable convergence. To prevent overfitting, an early stopping mechanism was implemented, which terminates training if the validation loss fails to improve for 10 consecutive epochs.

Due to significant class imbalance across functionalities, with classes such as antiviral and anticancer being relatively underrepresented, focal loss was used as the objective function with a parameter of $a_{t}=1$ and a focusing parameter of γ=2. Focal loss dynamically down-weights well-classified, easy examples and emphasizes harder, misclassified samples, effectively improving the model’s ability to learn from underrepresented classes. This strategy results in more balanced and accurate predictions across all five AMP functionalities.

**Figure 1:**
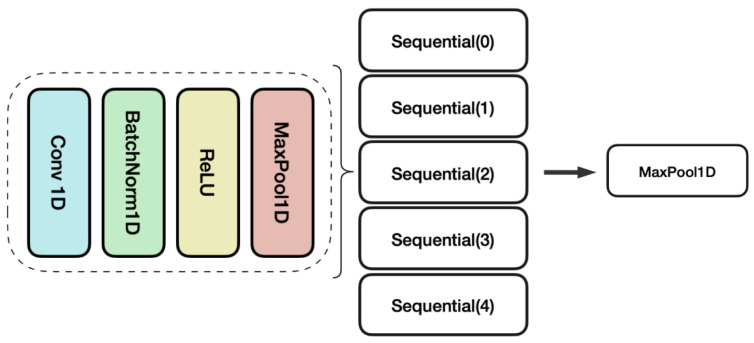
Layer configuration of multifunction prediction.

As illustrated in Figure 1, The model architecture consisted of 20 1D convolutional layers, structured into four sequential blocks. Each block contained a 1D convolutional layer, batch normalization, ReLU activation, max pooling, and an output. These layers progressively extracted hierarchical patterns from the input sequence encodings. After pattern extraction by the convolutional layers, the model employed a 257-unit fully connected layer to project the high-dimensional features into a lower-dimensional representation space. The final output layer used a sigmoid activation function to independently predict the probability of the five functional activity labels.

To evaluate the model performance, we used the independent test set to calculate standard classification metrics, including precision, recall, F1-score, sensitivity, specificity, and overall accuracy. These metrics provide a comprehensive view of the model’s effectiveness in predicting the diverse functional roles of AMPs.

Additionally, we conducted 10-fold stratified cross-validation to further assess the robustness and generalizability of the model. The dataset was divided into 10 balanced folds, maintaining consistent proportions of positive and negative samples across all functions. In each iteration, one fold was used for validation, while the remaining nine were used for training. This process ensured that every data point was evaluated during both training and testing, yielding a reliable estimate of real-world performance. Table 2 displays the prediction results across five functional categories. The training data column shows function-specific counts, where peptides with multiple functions are included in each relevant category. These counts represent unduplicated entries within each functional group, but since the same peptide may belong to multiple groups, the sum of function-specific counts exceeds the total unique peptide count in the AMP column. This reflects our evaluation approach, where functional annotations are considered separately from the complete peptide set.

### 2.2. Experimental Results

#### 2.2.1. Comparison of AMP Identification Models

In this section, we present a comparative analysis of the performance of various AMP classification models, including our proposed method. While classification was not the main objective of this study, it serves as a benchmark to assess our model’s performance against other machine learning-based AMP classifiers. It is important to note that our focus in this comparison is not on the differences between various machine learning algorithms, but rather, on evaluating the classification effectiveness.

The performance of each model was evaluated based on several key metrics: sensitivity (SEN), specificity (SPE), accuracy (ACC), precision (PRE), and Matthews Correlation Coefficient (MCC). These metrics provide a comprehensive assessment of the models’ ability to correctly identify AMPs and distinguish them from non-AMPs.

As shown in Table 3, our model achieves the highest SEN among all of the compared methods, with a value of 0.962, indicating a strong ability to correctly identify true AMPs with minimal false negatives. This performance result reflects our model’s design priority, maximizing coverage of potential AMP candidates through a regression-based MIC predictor, which favors recall over strict precision. Although AMPScannerV2 outperforms the other models in most other metrics, including SPE and precision, it yields a lower SEN (0.924), suggesting a higher likelihood of missing active peptides.

While our model’s specificity (0.862) is slightly lower than that of some alternatives, it still demonstrates a strong capability for filtering out non-AMPs. Its overall ACC of 0.912 remains competitive with the best-performing models. These results highlight the trade-off we adopt to reduce false negatives, which is critical in early-stage AMP discovery. Prioritizing sensitivity ensures that potential sequences are retained for further evaluation and reduces the risk of discarding valuable candidates.

#### 2.2.2. Analysis of the Quality and Multifunctionality of the Generated AMP Sequences

##### Multifunction Predictions

**Figure 2:**
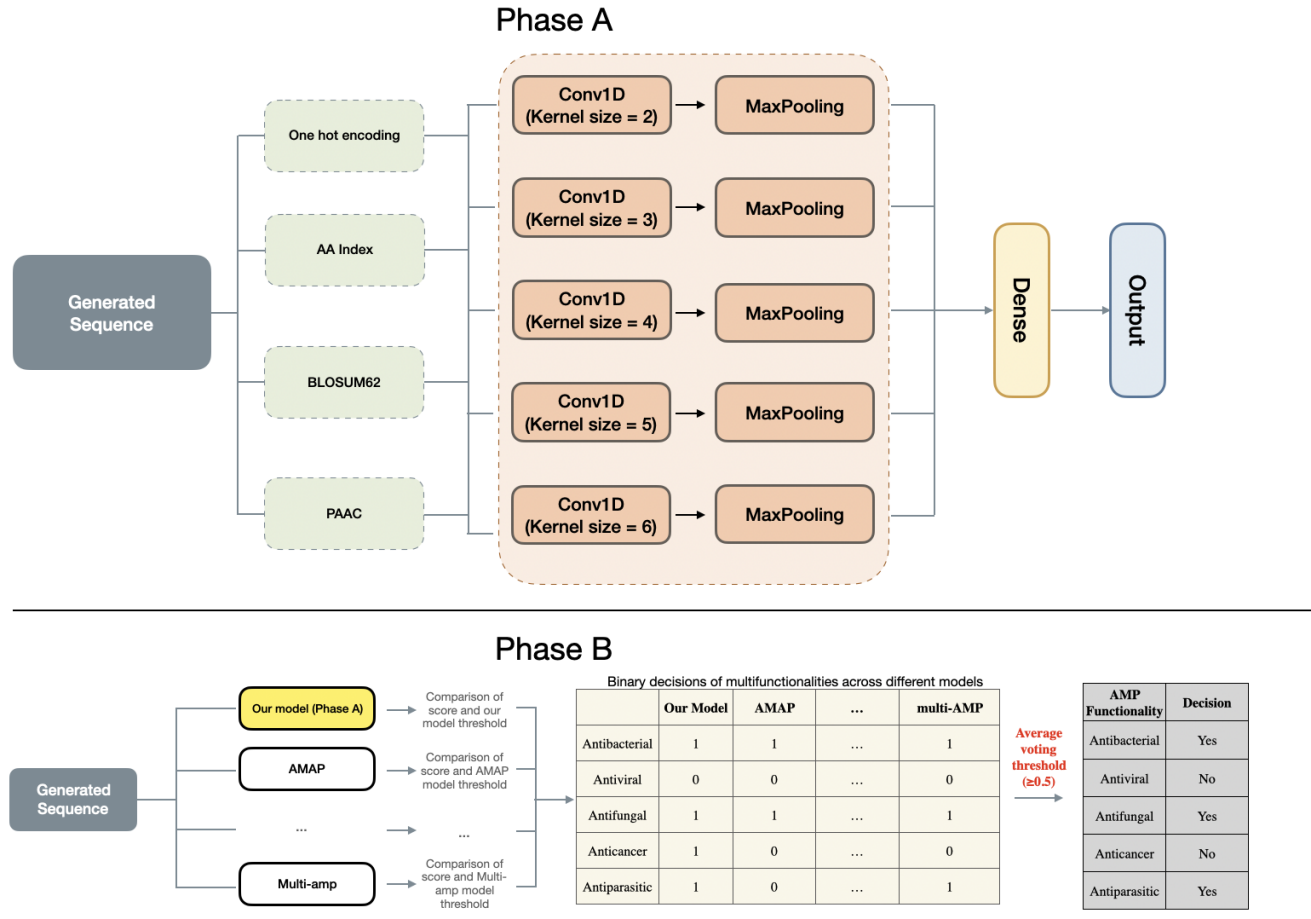
The architecture of the multifunction predictor. (**Phase A**): Our model uses four distinct encoding methods to independently process AMP sequences, with their embeddings fused through neural network integration; the aggregated features then pass through fully connected layers to simultaneously predict all five functional attributes. (**Phase B**): The system incorporates predictions from established AMP classifiers (AMAP, multi-AMP, etc.), where each model applies its own predefined threshold to convert scores to binary decisions before majority voting determines the resulting multifunction profile. This dual-phase design combines deep feature learning with robust cross-model validation.

**Figure 3:**
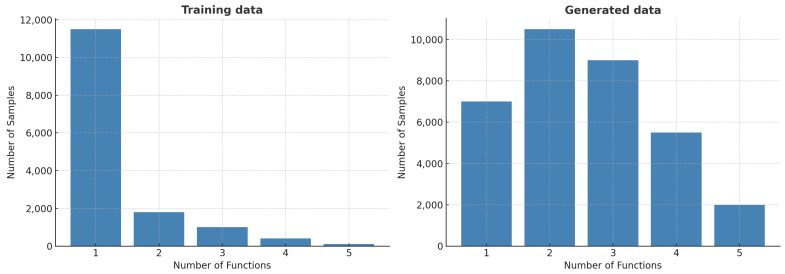
The distribution of multifunctional AMP sequences. A total of 5 functional activities were predicted for the generated AMPs, and this distribution is compared with that of the training dataset.

Our generative model produced a total of 139,389 AMP sequences. Among these, 35,448 were predicted to be positive AMPs, as illustrated in Figure 2, while the remaining 103,941 were classified as negative. We then performed multifunctional prediction on the positive AMP sequences to assess their functional diversity. Figure 3 compares the number of predicted functions per sequence between the training dataset and the generated dataset. In the training data, most AMPs possess only one function, with a steep drop-off beyond two functions. In contrast, the generated AMPs display a diverse functional distribution, with most sequences predicted to possess two or three functions. A smaller number exhibit broader multifunctionality, with some predicted to have all five functional activities.

This comparison highlights a key strength of our framework: its ability to generate AMPs with enhanced multifunctionality relative to the training dataset. All generated AMPs were predicted to possess at least one of the five target functions, with a substantial proportion exhibiting two or three activities (Figure 3). A smaller subset even demonstrated all five, reflecting the model’s capacity to integrate and recombine diverse functional patterns learned from the training data. As shown in Table 2, the predominantly predicted activity was anticancer (21,664), followed by antibacterial (17,288), antiviral (16,748), antiparasitic (14,348), and antifungal (13,431). This distribution illustrates the model’s ability to generate both broadly active and functionally specialized AMPs, underscoring its utility for multifunctional peptide design.

Additionally, Table 4 presents the detailed performance metrics of the five independently trained functional classifiers, providing quantitative evidence for the reliability of our multifunction prediction module. For each functional category, the classifier achieved strong performance: for example, antiviral activity prediction yielded the highest accuracy (0.9403) and AUC (0.9758), reflecting robust generalization to this functional type. Antiparasitic prediction also showed excellent results, with an F1-score of 0.9261 and MCC of 0.8518, demonstrating the model’s effectiveness in capturing sequence patterns associated with parasitic targeting. For antibacterial (F1 = 0.8750) and antifungal (F1 = 0.8731) activities, the classifiers maintained consistent performance, while anticancer prediction achieved a balanced F1-score of 0.8926, indicating reliable identification of peptides with tumor-targeting potential. These metrics collectively validate that our multi-hybrid embedding approach and CNN architecture effectively capture the diverse sequence features underlying different AMP functionalities, supporting the credibility of subsequent multifunctional candidate selection.

##### Physicochemical Properties

**Figure 4:**
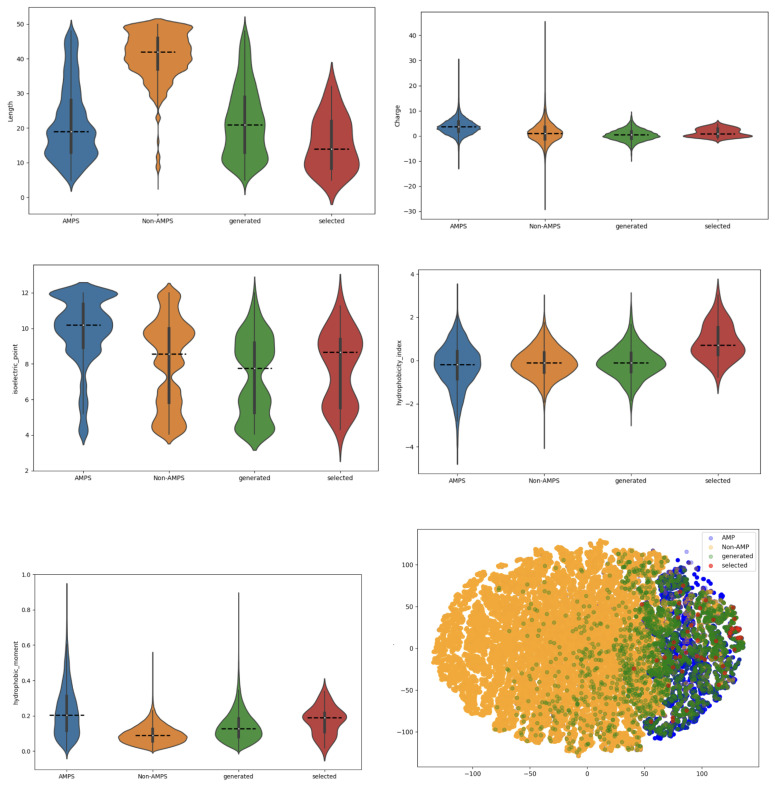
The images should be read from top to bottom. The first image presents the length of the peptides. The second image illustrates the charge, followed by the isoelectric point in the top-right image. The second row begins with the hydrophobicity index, followed by the hydrophobic moment, and the final image displays the T-SNE projection. The color blue represents real AMPs, orange represents non-AMPs, green represents AMPs generated by our model, and red represents the top 50 AMP sequences with the most functions.

As shown in Figure 4, we analyzed and compared the physicochemical properties of real AMPs, non-AMPs, generated AMPs, and the top 50 peptides with the highest number of predicted functions. The evaluated features included sequence length, net charge, isoelectric point, hydrophobicity index, hydrophobic moment, and a t-SNE projection derived from these properties.

The generated AMPs exhibit broader distributions than real AMPs across most properties. In particular, for isoelectric point, hydrophobicity index, and hydrophobic moment, the distributions of both the general generated set and the top multifunctional peptides appear closer to those of non-AMPs than canonical AMPs. This deviation is expected, as the FBGAN framework relies exclusively on regression-based MIC feedback and does not explicitly constrain physicochemical features during generation. However, for other properties, such as net charge and sequence length, the top-performing peptides show distributions more aligned with real AMPs. Furthermore, the t-SNE projection reveals that many of the top 50 sequences cluster near real AMP regions, suggesting that the model still captures essential AMP-like characteristics relevant to biological activity, even without physicochemical guidance.

#### 2.2.3. Ranking Generated AMP Sequences Based on Structural Confidence

To prioritize the generated AMP sequences for downstream validation, we employed a ranking strategy that incorporated multiple predictive metrics, with particular emphasis on the structural confidence scores generated by AlphaFold2. Specifically, we used the per-residue predicted Local Distance Difference Test (pLDDT) scores visualized in the IDDT line chart output by AlphaFold2 to assess the confidence of each predicted structure. These scores quantify the positional certainty of amino acid residues within the predicted fold, where higher values reflect greater structural reliability.

Sequences with consistently high pLDDT scores are considered more likely to adopt stable, well-defined conformations and are therefore prioritized for further biochemical characterization. This provides a practical and scalable approach to filtering large sets of generated candidates.

**Figure 5:**
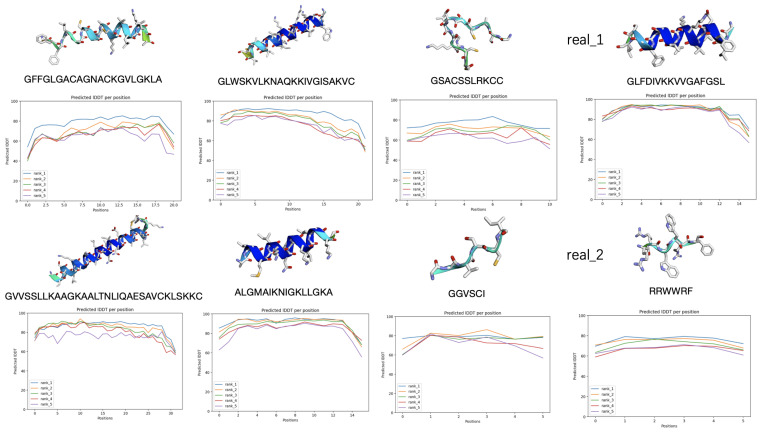
The ranking of the generated AMP sequences based on multiple prediction metrics, with particular emphasis on the structural confidence provided by AlphaFold2.0.

Figure 5 illustrates how this prioritization is implemented by comparing structural predictions and pLDDT profiles across several sequences. Real AMP examples, labeled as real_1 and real_2, demonstrate distinct structural features with varying confidence levels, as observed in both their three-dimensional models and residue-wise pLDDT plots. In contrast, several generated peptides display high-confidence alpha-helical conformations, with uniformly elevated pLDDT scores. Conversely, sequences with low or fluctuating confidence values, often suggestive of intrinsic disorder, are deprioritized. Integrating these structural confidence metrics into the ranking pipeline enables the informed, efficient selection of candidates most suitable for experimental validation.

## 3. Materials and Methods

### 3.1. Overall Framework of FBGAN-Based Model

**Figure 6:**
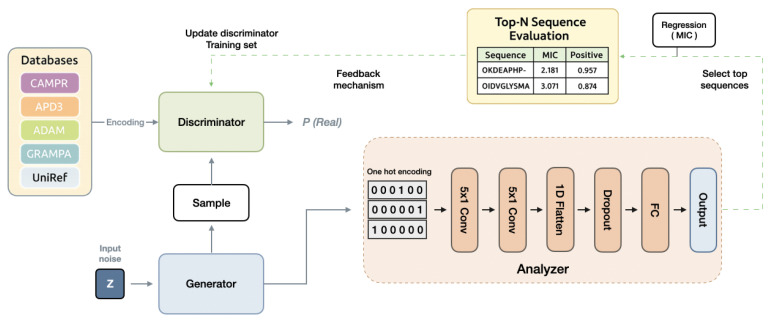
The FBGAN architecture functions as follows: During each epoch, the analyzer acquires a specific number of sequences from the generator. Following evaluation, the analyzer selects the top n sequences to refresh the discriminator’s training set, replacing the oldest n sequences. This iterative process occurs in every epoch, progressively enhancing the discriminator’s dataset with top-scoring sequences. Consequently, the model gradually shifts towards generating AMP sequences that exhibit the desired attributes.

For efficient AMP generation, we implement a feedback-loop mechanism [12] to enhance the GAN, as illustrated in Figure 6. The GAN model is utilized to create initial AMP sequences that lack desired attributes in the initial phase. An analyzer is employed to evaluate the generated AMP sequences from the generator and provide scores. We employ a Convolutional Neural Network (CNN) to predict the MIC value of AMPs and the likelihood of an AMP being effective, integrating these metrics to calculate the score. In each epoch, the analyzer receives a fixed number of sequences generated by the generator. It assesses these sequences, selecting the top-scoring *n* sequences to update the training set of the discriminator, replacing the *n* oldest sequences. Subsequently, the GAN undergoes standard training for one epoch. As training progresses, the discriminator’s entire training set is continuously refreshed with high-scoring generated sequences from the analyzer, gradually achieving the generation of AMP sequences with desired properties.

### 3.2. GAN Model Architecture

In this work, we adopt the GAN architecture to generate initial AMP sequences [41]. A GAN consists of two parts: a generator *G* that creates sequences from random noise, and a discriminator *D* that tries to tell whether a sequence is real or generated. G aims to produce sequences that resemble real data, while D learns to distinguish between real and synthetic sequences. The optimization of *G* and *D* is guided by the following loss function [41]:

(1) $$\begin{array}{cc} \min_{G}\max_{D}V\left(D,G\right)= & E_{x∼P_{\mathrm{data}}\left(x\right)}\left[\log D(x)\right]\\ & +E_{z∼P(z)}\left[\log\left(1-D(G(z))\right)\right] \end{array}$$

To improve training stability and sample quality, we adopt the Wasserstein GAN (WGAN) framework with a gradient penalty, as proposed by Gulrajani et al. [42].

### 3.3. AMP Activity Predictor

The generated AMP sequences are evaluated using a deep learning-based predictor [13], which predicts their antimicrobial activity. This predictor operates both as a regression model to predict the MIC and as a classifier to determine whether the sequence possesses antimicrobial properties.

The predictor employs a CNN architecture to simultaneously handle both classification and regression tasks, extracting features from peptide sequences encoded using one-hot encoding. In this encoding, each amino acid is represented as a binary vector. These encoded sequences are passed through shared convolutional and pooling layers, which capture hierarchical patterns and extract features indicative of antimicrobial activity. After the sequences are passed through these shared layers, the network branches into two separate output heads. The classification head consists of a dense layer followed by a sigmoid activation function, producing a probability value between 0 and 1 to indicate whether the peptide is antimicrobial; on the other hand the regression head includes a dense layer that outputs a single scalar value, representing the predicted MIC for the peptide. This dual-head architecture allows the model to effectively leverage shared features while specializing in both classification and regression tasks.

For the training, the model uses the same data for both tasks, where the input peptide sequences are shared between the classification and regression tasks. However, the target labels differ: in classification, the target is a binary label (0 or 1) indicating whether the peptide has antimicrobial properties, while in regression, the target is the actual MIC value, predicting the antimicrobial effectiveness of the peptide. By leveraging the same training data for both tasks, the model can learn representations that benefit both the classification and regression tasks efficiently. During training, the model minimizes a combined loss function, which includes both the binary classification loss and the regression loss, enabling it to simultaneously predict whether a peptide is antimicrobial and its corresponding MIC value. The model is trained using the Adam optimizer, minimizing the Mean Squared Error (MSE) between the predicted and actual MIC values, while also optimizing the classification loss using binary cross-entropy. By minimizing a combined loss function, the model learns to predict both the antimicrobial properties and MIC values effectively.

The predictor architecture begins with zero padding to standardize the input sequence dimensions, followed by 1D convolutional layers to capture sequence motifs and patterns indicative of antimicrobial properties. A max pooling layer reduces the dimensionality of the feature maps while retaining essential features. The output is then flattened into a 1D vector and passed through dense layers to learn complex relationships. A dropout layer is included to mitigate overfitting, and the final output layer predicts MIC values while supporting antimicrobial classification.

### 3.4. AMP Multifunction Predictor

To generate AMP sequences with multiple functions, we employ a multi-hybrid embedding approach inspired by the work [38], as depicted in Figure 2. AMPs exhibit a broad spectrum of biological activities, including antibacterial, antifungal, antiviral, antiparasitic and anticancer activities. Our objective is to identify AMPs that exhibit multiple functions simultaneously. To effectively capture the peptide characteristics, we utilize four different encoding techniques.

These four different types of amino acid information, representing peptide sequences, include one-hot, AAindex [43], BLOSUM62 [44], and PAAC encoding [45]. One-hot encoding represents each amino acid in the peptide sequence as a binary vector of length 20, corresponding to the 20 standard amino acids. Each position in the vector is filled with zeros, except for a single ‘1’ at the index representing the specific amino acid, this encoding allows for clear sequence representation in computational models. AAindex encoding maps each amino acid to a vector of real-valued features from the AAindex database, which contains numerical indices that capture the physicochemical properties of amino acids, such as hydrophobicity, polarity, and charge. This encoding provides a more detailed representation of the chemical and physical properties of peptides. BLOSUM62 matrix encoding uses a substitution matrix to capture evolutionary relationships between amino acids. Each amino acid is represented by a 20-dimensional vector, with each element corresponding to the substitution score of that amino acid against others in the matrix. We choose BLOSUM62 because it effectively balances sensitivity and specificity in capturing amino acid substitution patterns, which is crucial for predicting AMP activity. While other matrices exist, BLOSUM62 is widely preferred in bioinformatics for its ability to model evolutionary conservation in protein sequences. PAAC encoding combines the amino acid composition with the correlation factor between the physicochemical properties of amino acids. This method enhances the peptide representation by considering both the frequency of amino acids and their interactions within the peptide. PAAC encoding has been shown to improve prediction accuracy for tasks involving peptide function, especially when predicting the biological properties of AMPs.

These four encoding techniques capture different aspects of the peptide sequence, creating a richer, more informative feature set. After extracting these features, they are concatenated into a unified feature vector, which is then passed through the model for subsequent AMP function prediction tasks. This multi-hybrid embedding approach allows the model to leverage diverse sources of information and enhance the overall prediction of multiple biological activities exhibited by the generated AMPs. The encoded AMP sequences are then fed into a CNN. The embeddings are fused into a single feature representation, which is subsequently passed through a fully connected layer to predict multiple attributes, including antibacterial, antiviral, antifungal, anticancer, and antiparasitic activities.

The framework integrates multiple AMP function prediction models, including IAMPCN [19], AMAP [46], CAMP [25], iamp-2l [26], iamp-CA2L [27], and multi-AMP [47] enabling the prediction of 5 different AMP functionalities. The voting process integrates predictions from 6 pre-trained models to determine the multifunctional activities of AMPs, where each model applies a unique probability threshold for classifying specific functionalities. First, each model produces a prediction score that is compared against its specific threshold, yielding binary labels (1 for present, 0 for absent) for each functionality. These labels are then aggregated into a table, where the mean value of binary outputs per functionality is calculated. A horizontal decision threshold of 0.5 determines the final classification: if the mean exceeds this value, the peptide is deemed to possess the functionality. This integration method harmonizes predictions across diverse models to enhance the reliability of functionality determination.

## 4. Conclusions

The escalating threat of antimicrobial resistance has necessitated the development of innovative strategies to combat multi-drug-resistant infections. This study presents a novel de novo multifunctional AMP design framework that leverages a hybrid generative approach, integrating an FBGAN with a global quantitative AMP activity regression module and a multifunctional-attribute integrated prediction module. Our framework addresses the critical need for high-potential multifunctional AMPs, offering a potent tool in the arsenal against microbial resistance.

Through extensive computational evaluation, we demonstrated the capability of our generative framework to design novel AMPs with both strong predicted antimicrobial potency and broad-spectrum functional potential. Peptides exhibiting multiple enhanced antimicrobial properties are those identified by our framework as having low predicted MIC values through the FBGAN analyzer regression module, while simultaneously being assigned multiple functional activities via our multi-label classification models. This indicates potential efficacy across a wide range of pathogens.

A key strength of our approach lies in the integration of comprehensive and updated AMP databases with an advanced multi-task prediction strategy. This allowed the model to annotate generated sequences with five distinct functional attributes, offering a detailed and multifaceted profile of their potential biological activity. By jointly optimizing for both potency and multifunctionality, the framework provides an effective and scalable means to prioritize promising AMP candidates from a vast and largely unexplored peptide sequence space.

This comparison of physicochemical properties between generated AMPs and real AMP sequences revealed a striking similarity, further validating the reliability of our model. The top 50 AMP sequences, selected for their multifunctionality, showed physicochemical properties most closely aligned with real AMPs, indicating the high quality and potential usage of our generated candidates.

By integrating curated AMP databases, multi-label functional prediction models, and structural plausibility screening via AlphaFold2, our framework prioritized peptides with promising characteristics, such as low predicted MIC values and diverse biological functions. From over 139,000 generated sequences, the model identified 35,448 candidates with predicted AMP activity and selected the top 50 multifunctional sequences for further consideration (as presented in Table 2 and Figure 4). This computational strategy aligns with established practices in drug discovery, where in silico modeling and candidate prioritization precede experimental synthesis and validation.

In the future we plan to experimentally validate our computational predictions, and we will collaborate with partner laboratories to characterize top-ranked multifunctional peptide candidates. Selected peptides will undergo (1) chemical synthesis and purity analysis, (2) antimicrobial susceptibility testing (MIC/MBC) against clinically relevant pathogens, and (3) functional validation of predicted activities (antibacterial, antifungal, anticancer, antiviral, antiparasitic) using standardized assays. All results will be benchmarked against established AMP controls. This collaborative effort will both verify our predictive framework and provide experimental data to refine the FBGAN generator and multi-label classifier through iterative improvement cycles.

## Figures and Tables

**Table 1:** Mapping of 22 original functional labels to 5 core categories.

Core Functional Categories	Original Labels Included
Antibacterial	Antibacterial, anti-Gram-positive, Anti-Gram-negative, anti-TB, antibiofilm
Antifungal	Antifungal, anticandida
Anticancer	Anticancer, anti-mammalian-cell
Antiviral	Antiviral, anti-HIV
Antiparasitic	Antiparasitic, antimalarial, antiplasmodial, antiprotozoal

**Table 2:** Number of positive and negative samples for AMP functional prediction and training.

Functions	Classification Results		Training Dataset	
	Positive	Negative	Positive	Negative
AMPs	35,448	103,941	14,731	19,793
Antibacterial	17,288	18,160	8865	7865
Antifungal	13,431	22,017	3926	12,993
Antiviral	16,748	18,700	3525	13,384
Anticancer	21,664	13,784	3262	13,630
Antiparasitic	14,348	21,100	313	16,287

**Table 3:** Performance comparison of different AMP identification models.

Identification Task					
Model	SEN ↑	SPE ↑	ACC ↑	PRE ↑	MCC ↑
CAMP-SVM [25]	0.826	0.870	0.848	0.864	0.696
CAMP-RF [25]	0.876	0.926	0.901	0.922	0.803
CAMP-ANN [25]	0.852	0.854	0.853	0.853	0.705
CAMP-DA [25]	0.876	0.902	0.889	0.899	0.778
diff-AMP [38]	0.830	0.914	0.869	0.915	0.741
iAMPpred [39]	0.860	0.887	0.873	0.885	0.747
AMPscannerv2 [40]	0.924	**0.928**	**0.926**	**0.928**	**0.852**
ours	**0.962**	0.862	0.912	0.874	0.828

**Table 4:** Classification performance of five independently trained AMP functional classifiers.

Performance of Function-Specific AMP Classifiers					
Functions	Accuracy ↑	Precision ↑	F1-Score ↑	MCC ↑	AUC↑
Antibacterial	0.8722	0.8706	0.8750	0.7445	0.9445
Antifungal	0.8763	0.8745	0.8731	0.7429	0.9423
Antiviral	0.9403	0.9446	0.9408	0.8806	0.9758
Anticancer	0.8934	0.9014	0.8926	0.7865	0.9561
Antiparasitic	0.9256	0.9221	0.9261	0.8518	0.9683

## Data Availability

The data presented in this study are available on request from the corresponding author.

## Abbreviations

The following abbreviations are used in this manuscript:

AMPs	Antimicrobial peptides
FBGAN	Feedback Generative Adversarial Network
GAN	Generative Adversarial Network
VAE	Variational Autoencoder
MIC	Minimum Inhibitory Concentrations
APD3	Antimicrobial Peptide Database
DRAMP	Data Repository of Antimicrobial Peptides
DNN	Deep Neural Network
CNN	Convolutional Neural Network
MSE	Mean Squared Error
GRAMPA	Giant Repository of AMP Activities
CAMPR4	Collection of Anti-Microbial Peptides
SEN	Sensitivity
SPE	Specificity
ACC	Accuracy
PRE	Precision
MCC	Matthews Correlation Coefficient

## References

[1] D’Costa V.M., King C.E., Kalan L., Morar M., Sung W.W., Schwarz C., Froese D., Zazula G., Calmels F., Debruyne R. (2011). Antibiotic resistance is ancient. Nature.

[2] Gaspar D., Veiga A.S., Castanho M.A.R.B. (2013). From antimicrobial to anticancer peptides: A review. Front. Microbiol..

[3] Magana M., Pushpanathan M., Santos A.L., Leanse L., Fernandez M., Ioannidis A., Giulianotti M.A., Apidianakis Y., Bradfute S., Ferguson A.L. (2020). The value of antimicrobial peptides in the age of resistance. Lancet Infect. Dis..

[4] Lei J., Sun L., Huang S., Zhu C., Li P., He J., Mackey V., Coy D.H., He Q. (2019). The antimicrobial peptides and their potential clinical applications. Am. J. Transl. Res..

[5] Wan F., Wong F., Collins J.J., Fuente-Nunez C.D. (2024). Machine learning for antimicrobial peptide identification and design. Nat. Rev. Bioeng..

[6] Das P., Sercu T., Wadhawan K., Padhi I., Gehrmann S., Cipcigan F., Chenthamarakshan V., Strobelt H., Santos C.D., Chen P. (2021). Accelerated antimicrobial discovery via deep generative models and molecular dynamics simulations. Nat. Biomed. Eng..

[7] Tucs A., Tran D.P., Yumoto A., Ito Y., Uzawa T., Tsuda K. (2020). Generating ampicillin-level antimicrobial peptides with activity-aware generative adversarial networks. ACS Omega.

[8] Oort C.M.V., Ferrell J.B., Remington J.M., Wshah S., Li J. (2021). AMPGAN v2: Machine learning-guided design of antimicrobial peptides. J. Chem. Inf. Model..

[9] Surana S., Arora P., Singh D., Sahasrabuddhe D., Valadi J. (2023). PandoraGAN: Generating antiviral peptides using generative adversarial network. SN Comput. Sci..

[10] Dean S.N., Alvarez J.A.E., Zabetakis D., Walper S.A., Malanoski A.P. (2021). PepVAE: Variational autoencoder framework for antimicrobial peptide generation and activity prediction. Front. Microbiol..

[11] Lazzaro B.P., Zasloff M., Rolff J. (2020). Antimicrobial peptides: Application informed by evolution. Science.

[12] Gupta A., Zou J. (2018). Feedback GAN (FBGAN) for DNA: A novel feedback-loop architecture for optimizing protein functions. arXiv.

[13] Witten J., Witten Z. (2019). Deep learning regression model for antimicrobial peptide design. BioRxiv.

[14] Wang G., Li X., Wang Z. (2016). APD3: The antimicrobial peptide database as a tool for research and education. Nucleic Acids Res..

[15] Li J., Pu Y., Tang J., Zou Q., Guo F. (2020). DeepAVP: A dual-channel deep neural network for identifying variable-length antiviral peptides. IEEE J. Biomed. Health Inform..

[16] Sharma R., Shrivastava S., Singh S.K., Kumar A., Saxena S., Singh R.K. (2022). Deep-AFPPred: Identifying novel antifungal peptides using pre-trained embeddings from Seq2Vec with 1D-CNN-BiLSTM. Briefings Bioinform..

[17] Singh V., Shrivastava S., Singh S.K., Kumar A., Saxena S. (2022). StaBle-ABPpred: A stacked ensemble predictor based on biLSTM and attention mechanism for accelerated discovery of antibacterial peptides. Briefings Bioinform..

[18] Sharma R., Shrivastava S., Singh S.K., Kumar A., Saxena S., Singh R.K. (2021). Deep-ABPpred: Identifying antibacterial peptides in protein sequences using bidirectional LSTM with word2vec. Briefings Bioinform..

[19] Xu J., Li F., Li C., Guo X., Landersdorfer C., Shen H.H., Peleg A.Y., Li J., Imoto S., Yao J. (2023). IAMP-CN: A deep-learning approach for identifying antimicrobial peptides and their functional activities. Briefings Bioinform..

[20] Wang X.-F., Tang J.-Y., Liang H., Sun J., Dorje S., Peng B., Ji X.-W., Li Z., Zhang X.-E., Wang D.-B. (2024). ProT-Diff: A Modularized and Efficient Approach to De Novo Generation of Antimicrobial Peptide Sequences through Integration of Protein Language Model and Diffusion Model. bioRxiv.

[21] Tucs A., Berenger F., Yumoto A., Tamura R., Uzawa T., Tsuda K. (2023). Quantum Annealing Designs Nonhemolytic Antimicrobial Peptides in a Discrete Latent Space. ACS Med. Chem. Lett..

[22] Cai J., Yan J., Un C., Wang Y., Campbell-Valois F.-X., Siu S.W.I. (2025). BERT-AmPEP60: A BERT-Based Transfer Learning Approach to Predict the Minimum Inhibitory Concentrations of Antimicrobial Peptides for *Escherichia coli* and *Staphylococcus aureus*. J. Chem. Inf. Model..

[23] Lee H.T., Lee C.C., Yang J.R., Lai J.Z., Chang K.Y. (2015). A large-scale structural classification of antimicrobial peptides. Biomed. Res. Int..

[24] Gawde U., Chakraborty S., Waghu F.H., Barai R.S., Khanderkar A., Indraguru R., Shirsat T., Idicula-Thomas S. (2023). CAMPR4: A database of natural and synthetic antimicrobial peptides. Nucleic Acids Res..

[25] Thomas S., Karnik S., Barai R.S., Jayaraman V.K., Idicula-Thomas S. (2010). CAMP: A useful resource for research on antimicrobial peptides. Nucleic Acids Res..

[26] Xiao X., Wang P., Lin W.Z., Jia J.H., Chou K.C. (2013). IAMP-2L: A two-level multi-label classifier for identifying antimicrobial peptides and their functional types. Anal. Biochem..

[27] Xiao X., Shao Y.T., Cheng X., Stamatovic B. (2021). IAMP-CA2L: A new CNN-BiLSTM-SVM classifier based on cellular automata image for identifying antimicrobial peptides and their functional types. Briefings Bioinform..

[28] Chung C.R., Kuo T.R., Wu L.C., Lee T.Y., Horng J.T. (2020). Characterization and identification of antimicrobial peptides with different functional activities. Briefings Bioinform..

[29] Shi G., Kang X., Dong F., Liu Y., Zhu N., Hu Y., Xu H., Lao X., Zheng H. (2022). DRAMP 3.0: An enhanced comprehensive data repository of antimicrobial peptides. Nucleic Acids Res..

[30] Jhong J.-H., Chi Y.-H., Li W.-C., Lin T.-H., Huang K.-Y., Lee T.-Y. (2019). dbAMP: An integrated resource for exploring antimicrobial peptides with functional activities and physicochemical properties on transcriptome and proteome data. Nucleic Acids Res..

[31] Gogoladze G., Grigolava M., Vishnepolsky B., Chubinidze M., Duroux P., Lefranc M.-P., Pirtskhalava M. (2014). dbaasp: Database of antimicrobial activity and structure of peptides. FEMS Microbiol. Lett..

[32] Zhao X., Wu H., Lu H., Li G., Huang Q. (2013). LAMP: A Database Linking Antimicrobial Peptides. PLoS ONE.

[33] Mehta D., Anand P., Kumar V., Joshi A., Mathur D., Singh S., Tuknait A., Chaudhary K., Gautam S.K., Gautam A. (2014). ParaPep: A web resource for experimentally validated antiparasitic peptide sequences and their structures. Database.

[34] Hammami R., Hamida J.B., Vergoten G., Fliss I. (2008). PhytAMP: A database dedicated to antimicrobial plant peptides. Nucleic Acids Res..

[35] Qureshi A., Thakur N., Tandon H., Kumar M. (2013). AVPdb: A database of experimentally validated antiviral peptides targeting medically important viruses. Nucleic Acids Res..

[36] Tyagi A., Tuknait A., Anand P., Gupta S., Sharma M., Mathur D., Joshi A., Singh S., Gautam A., Raghava G.P.S. (2015). CancerPPD: A database of anticancer peptides and proteins. Nucleic Acids Res..

[37] Théolier J., Fliss I., Jean J., Hammami R. (2014). MilkAMP: A comprehensive database of antimicrobial peptides of dairy origin. Dairy Sci. Technol..

[38] Wang R., Wang T., Zhuo L., Wei J., Fu X., Zou Q., Yao X. (2024). Diff-AMP: Tailored designed antimicrobial peptide framework with all-in-one generation, identification, prediction and optimization. Briefings Bioinform..

[39] Meher P.K., Sahu T.K., Saini V., Rao A.R. (2017). Predicting antimicrobial peptides with improved accuracy by incorporating the compositional, physicochemical and structural features into Chou’s general PseAAC. Sci. Rep..

[40] Veltri D., Kamath U., Shehu A. (2018). Deep learning improves antimicrobial peptide recognition. Bioinformatics.

[41] Goodfellow I., Pouget-Abadie J., Mirza M., Xu B., Warde-Farley D., Ozair S., Courville A., Bengio Y. (2014). Generative adversarial nets. Advances in Neural Information Processing Systems.

[42] Arjovsky M., Chintala S., Bottou L. (2017). Wasserstein generative adversarial networks. International Conference on Machine Learning.

[43] Kawashima S., Ogata H., Kanehisa M. (1999). AAIndex: Amino acid index database. Nucleic Acids Res..

[44] Henikoff S., Henikoff J. (1992). Amino acid substitution matrices from protein blocks. Proc. Natl. Acad. Sci. USA.

[45] Chou K.C. (2009). Pseudo amino acid composition and its applications in bioinformatics, proteomics and system biology. Curr. Proteom..

[46] Gull S., Shamim N., Minhas F. (2019). AMAP: Hierarchical multi-label prediction of biologically active and antimicrobial peptides. Comput. Biol. Med..

[47] Qiaozhen M., Tang J., Guo F. Multi-AMP: Detecting the antimicrobial peptides and their activities using multi-task learning. Proceedings of the 2021 IEEE International Conference on Bioinformatics and Biomedicine (BIBM).

